# Supplementary material for: Nanopore sequencing reveals hidden landscape of short L1 transductions in colorectal cancer
Source: Commun Biol. 2026 Feb 12;9:418. doi: 10.1038/s42003-026-09674-z (PMC13009371; doi:10.1038/s42003-026-09674-z)
Supplement: Supplementary file 1 — Supplementary information [file 42003_2026_9674_MOESM1_ESM.pdf]

Supplementary information

Reference proxies for polymorphic L1 elements

The detection of 3’ transductions is complicated by the presence of proxy elements: truncated L1 insertions present in the reference genome that contain transduced sequence derived from a source element absent from the reference. Thus, the new offspring from the source element will be linked to the truncated element, as the proxy contains a more recognizable sequence for alignment. We identified two proxy elements in the reference GRCh38 (Supplementary information Table 1). Both elements were initially associated with transductions detected by xTea and Nanopore sequencing; however, identification of proxy elements allowed for correct reassignment of these transductions to their true sources.

Supplementary information table 1: Detected proxy elements in reference genome GRCh38 and their parents. The genomic positions are in GRCh38 coordinates. Number of somatic transductions detected initially from them with Nanopore and xTea are shown.

**Proxy id**	**Genomic position**	**Parent id**	**Parent position**	**Somatic insertions Nanopore**	**Somatic insertions xTea**
Yp11.2-3	chrY:9591746-9592012	6q25.3-1	chr6:157547705-157547718	10	25
19q13.31-1	chr19:44510544-44546504	6p24.1-1	chr6:13190785-1319080	73	6

The identification of proxy elements is highly dependent on the reference genome used. For example, the proxy element 19q13.31-1 (associated with source 6p24.1-1) is present in hg38 but absent in both hg37 and the T2T reference. However, a new insertion corresponding to this proxy is present in T2T at chr17:67,515,576. In contrast, proxy element Yp11.2-3 is found in both hg37 and T2T assemblies. Notably, neither 6q25.3-1 nor 6p24.1-1—the actual source elements—are present in any of the current reference genomes. This underscores the importance of analyzing L1 evolutionary lineages when assessing source activity. 6p24.1-1, for instance, emerges as the most somatically active element in our dataset, although the majority of transductions arising from it were initially misattributed to its proxy.

Targeted transduction validation

To validate both the tag and nanopore deduced transduction events, we performed PCR amplification and sequencing on a subset of somatic insertions (Methods: PCR and Sequencing). In total, 20 candidate insertions from each platform were selected. 10/20 in

Nanopore calls were detected with xTea as solo-L1s. Three types of primers were designed to amplify the inserted sequence (Supplementary Figure S1a): over-primers span the insertion breakpoint amplifying only sequence in the target that contains the insertion, tag-primers contain transduced sequence and out-primers containing only reference sequence. PCRs were performed with matched normal samples serving as negative controls. Using over-primers and pairing them with out-primers amplification was successful for 18/20 Nanopore detected and 11/20 tag-based insertions (Supplementary Data S5, Supplementary Data S3). However, Sanger sequencing was able to validate only the presence of insertion for most calls as the transduction sequence was inaccessible for Sanger sequencing due to flanking polyA-tails (Supplementary Figure S1b, Supplementary Data S5). However, by utilizing Nanopore sequencing (Methods: PCR and Sequencing) transductions were detected in 18/18 Nanopore WGS calls and 6/11 of short read tag based calls (Supplementary Figure S1b, Supplementary Data S5).

To directly access the transduced sequences we used tag-primers and out-primers (Supplementary Figure S1c) for 8/20 Nanopore and 12/20 tag detected transductions. 3/12 tag calls produced an unspecific result and 1/12 empty well, 8/12 produced a single band (Supplementary Data S5). However, we were able to successfully sequence only 2/8 of the tag based calls. With Nanopore detected elements we succeeded with 7/8 with one empty well, and we got the expected sequence from 4/7 (Supplementary Data S5).

While other insertions were validated to be somatic by performing the PCR in the same conditions in corresponding normal, one Illumina call produced a faint band in the tumor as is common for highly sensitive detection assays in colon tissue due to mosaic insertions prior to tumor expansion[1] (Supplementary Data S5).

To conclude, we were able to validate the presence of insertion in 18/20 Nanopore calls and 13/20 short read tag based calls. The presence of transductions was validated for 18/18 and 8/11 insertions. In transductions called by xTea as solo-L1 but with Nanopore as transductions we detected insertion and transduction in 10/10. These findings support that our calls detect true somatic transduction events and although the unique sequence was not detected with xTea in many of them, they were present in the insertion sequence.

Supplementary Figures

Supplementary Figure S1: Schematic of a partnered transduction

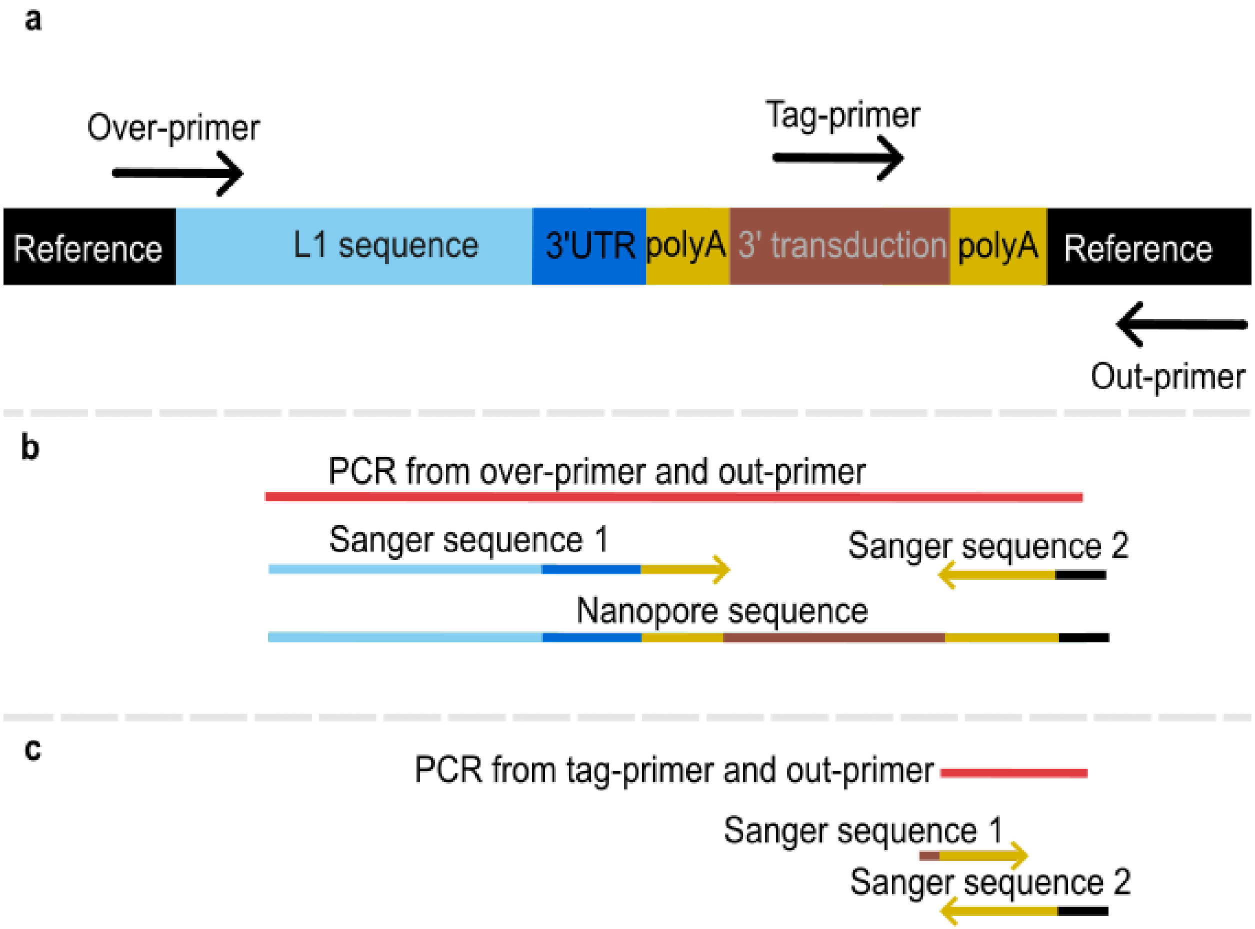

a) Schematic of a partnered transduction and designed primers PCR validation. The sequence consists of reference sequence flanking the insertion with L1 sequence with a 3´UTR and a polyA-tail of the source. Following the polyA-tail is the 3´ transduction and a polyA-tail resulting from the insertion. Three types of primers were designed to validate them: over-primers span the reference and insertion sequence, Tag-primers contain the 3´ transduction sequence and Out-primers contain only reference sequence outside the insertion. **b)** A PCR product from Over-primer and Out-primer will contain most of the insertion sequence and small amount of reference. Sanger sequencing of the product however, will be truncated by the polyA-tails flanking the transduction. Thus, sequencing starting from both ends of the strand can produce sequence supporting the insertion and its position in the reference, but not the transduction. When the same strand is sequenced with Nanopore, we will receive a whole sequence. **c)** A PCR product from Tag-primer and Out-primer will contain mainly polyA-tail with small amounts of reference and possibly transduction sequence. Sequencing of the primer will contain all components of the product if sequenced from both ends.

Supplementary Figure S2: Somatic activity of source L1s with Nanopore and tag selection

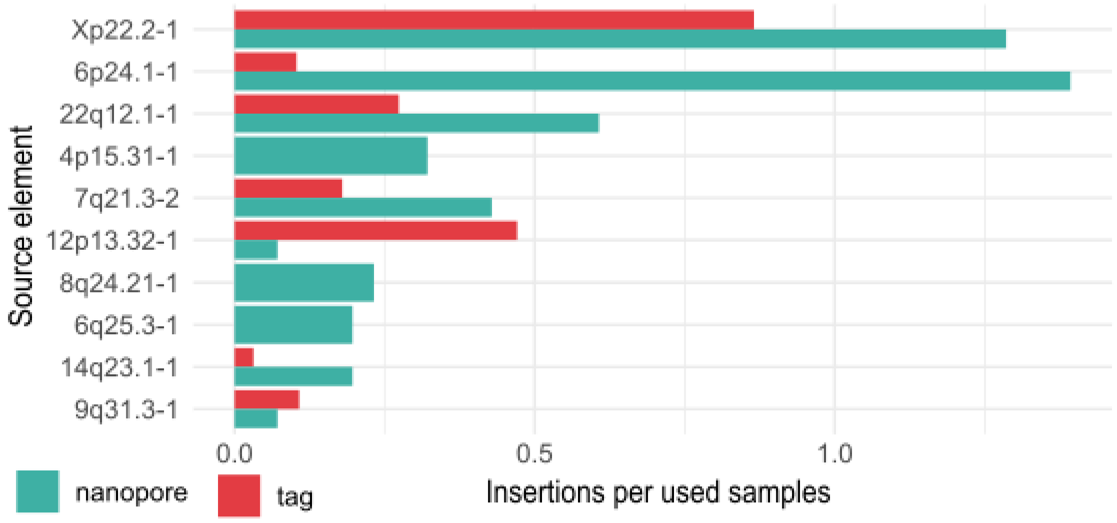

The frequency of somatic insertions arising from different source L1s in Nanopore detection vs. in tag selection. The figure presents ten most active sources. With tag-based detection, only transductions that unambiguously map to source elements are taken into account.

Supplementary Figure S3: Gel Images

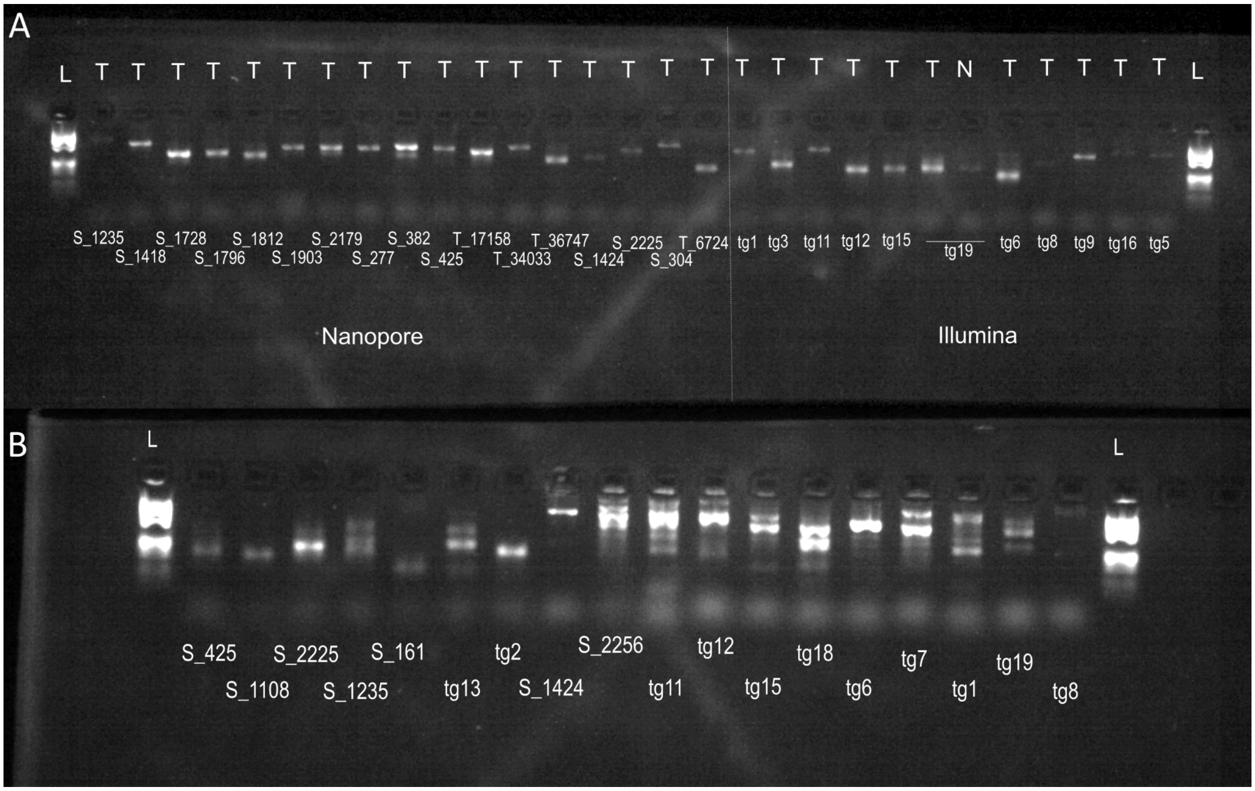

Agarose gel (3%) of somatic transductions producing a result in PCR, design specified in Supplementary Figure S1 A) Produced with over and out primer B) produced with tag primer and out primer. Annotation key: L (ladder; phi174 DNA-HaeIII marker), N (normal), T (tumor). Transduction loci specified in Supplementary Data S5.

Supplementary Figure S4: 5´breakpoints in transductions

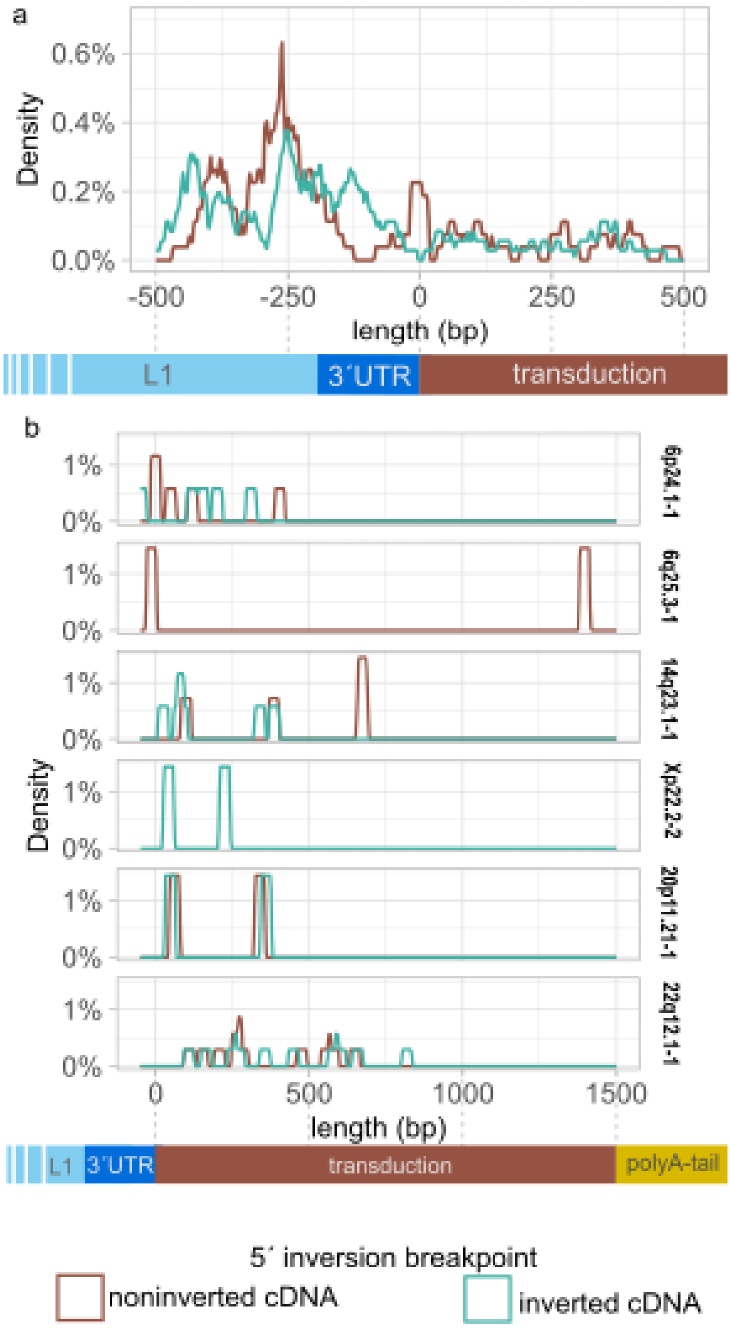

Rectangular kernels (bw = 10) presenting 5´inversion breakpoints detected in transductions. Breakpoints are separated by the cDNA where the breakpoints belong to. **a)** Inversion breakpoints 500 bp inside the 3´ end of L1 and 500 bp outside the element. **b)** Inversion breakpoints outside the element in source L1s where any breakpoints occur in the flanking sequence.

Supplementary Figure S5: Transduction end points with PAS

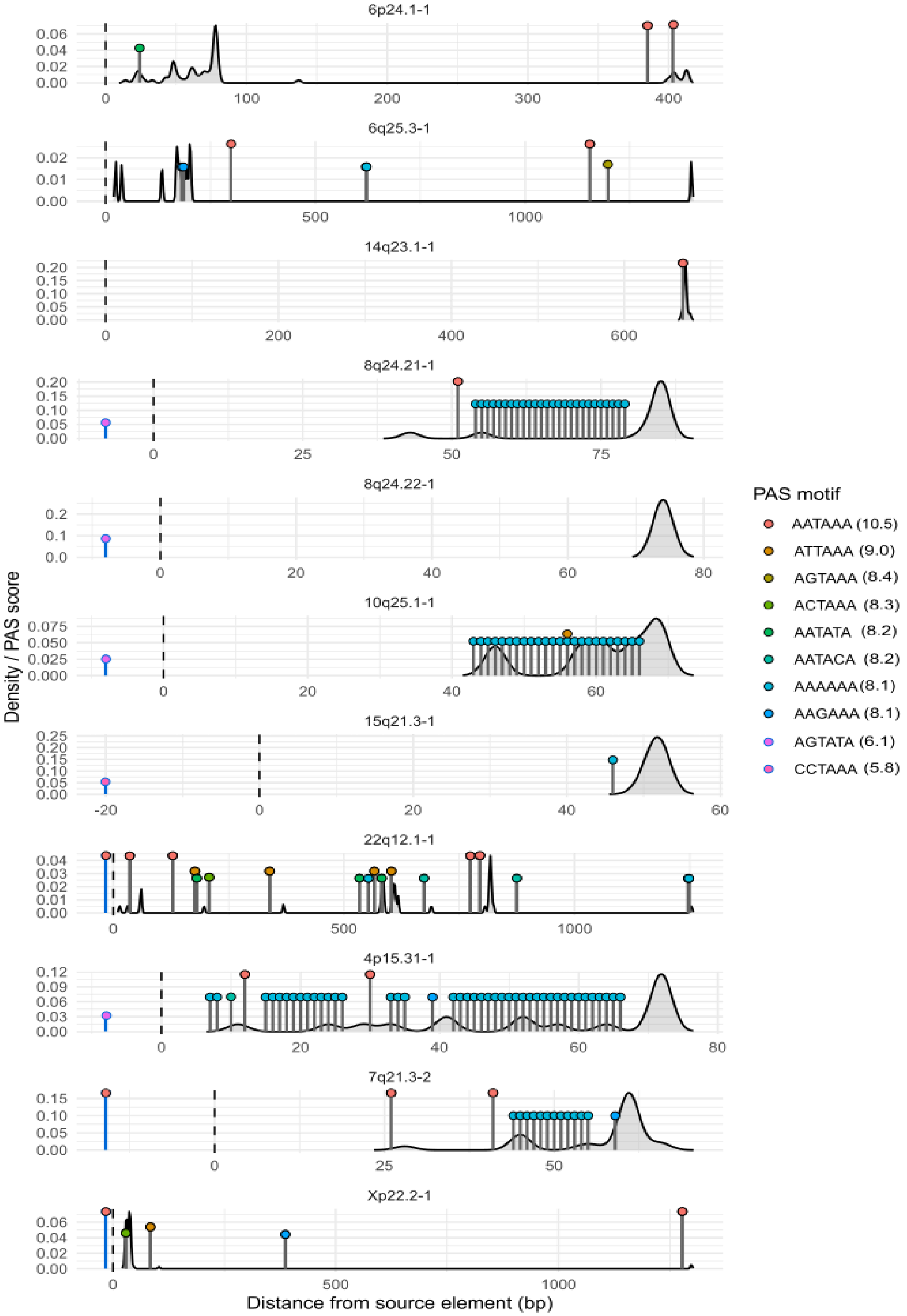

Density plot of transduction end points of sources elements with > 4 insertions in plot. Lollipops present the PAS with, where the height corresponds to the signal strength and color to the sequence. PAS downstream of source L1s are filtered by their score (score > 8.1). The reference elements have their internal PAS represented by a blue lollipop at the start of the plots.

Supplementary figure S6: Methylation levels at source L1 elements

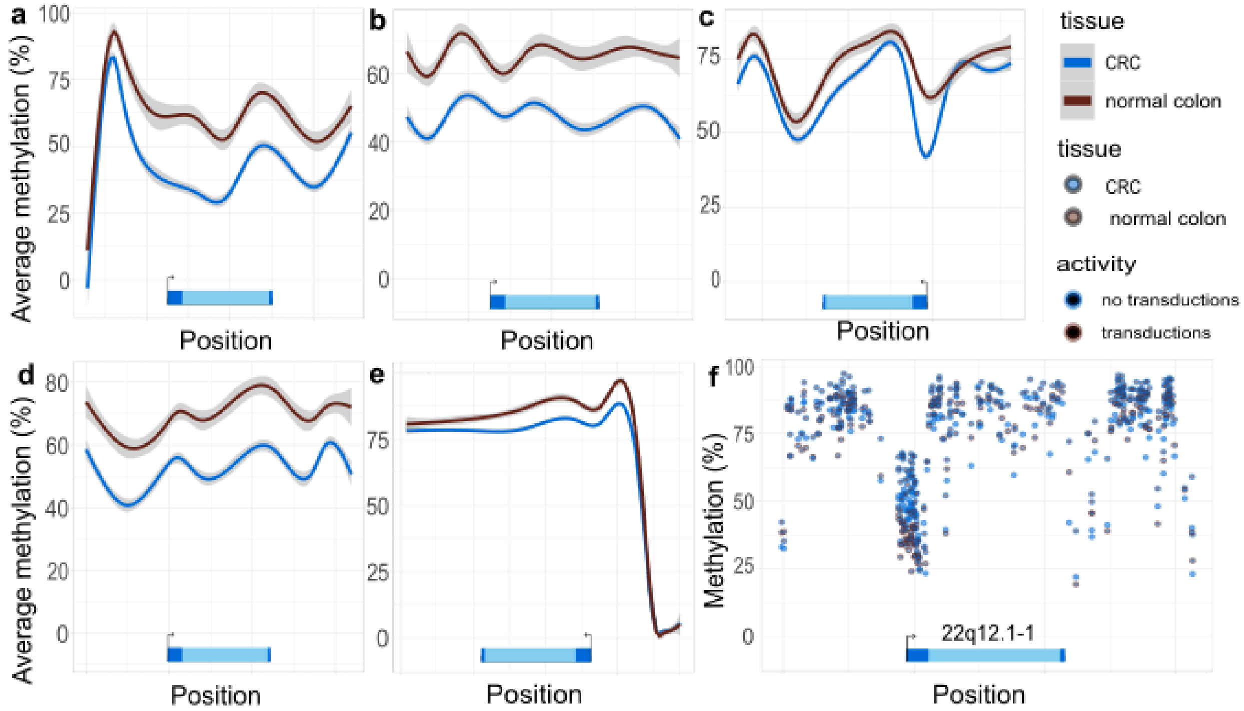

Smoothed methylation curves for 5 random inactive reference source L1 **a)** chrX:66553489-66559786 **b)** chr9:16294081-16300587 **c)** chr8:94003622-94009766 **d)** chr4:82175410-82181538 **e)** chr3:120732565-120738623 **f)** Average methylation values per tissue type (CRC or normal colon) are shown for individual CpGs around source L1 element 22q12.1-1.

Supplementary Figure S7: Phased methylation data around X-chromosomal source L1 element

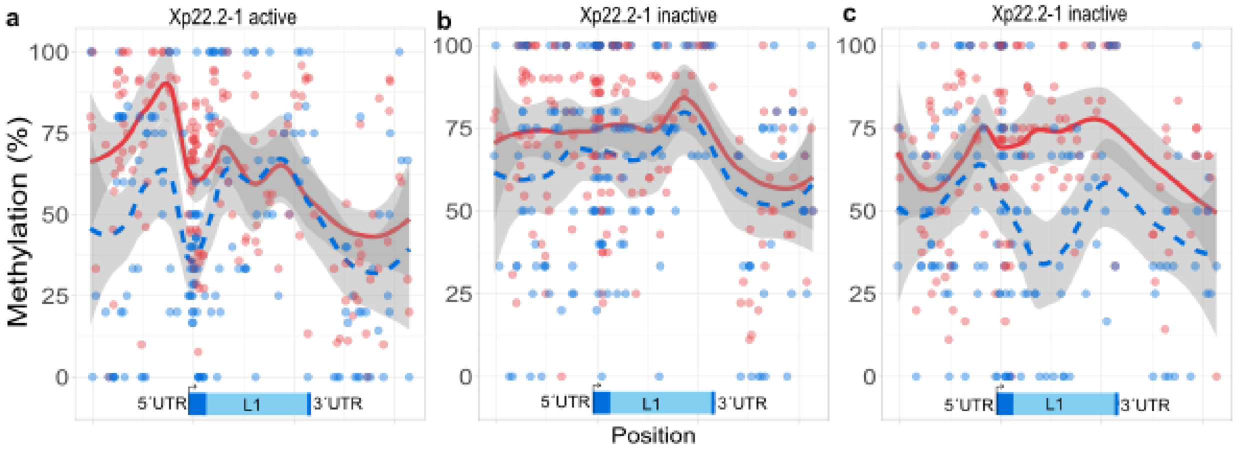

Phased methylation values around X-chromosomal Xp22.2-1 in women. Lines and colours depict data from two different alleles. Samples in the figures are **a)** c45_1_5259 with detected activity, **b**) c55_1_5391 with no activity and **c)** Fam_c589_1_8252TK with no activity. In **a)** methylation decreases in the other X-chromosome copy while this is not observed in b and c. In c, the drop in methylation is in another location, not near the promoter. This figure was created using ggplot2 function stat_smooth with method='loess'. Smoothing span was set to 0.5.

Supplementary Figure S8: Average methylation levels for individual L1 elements

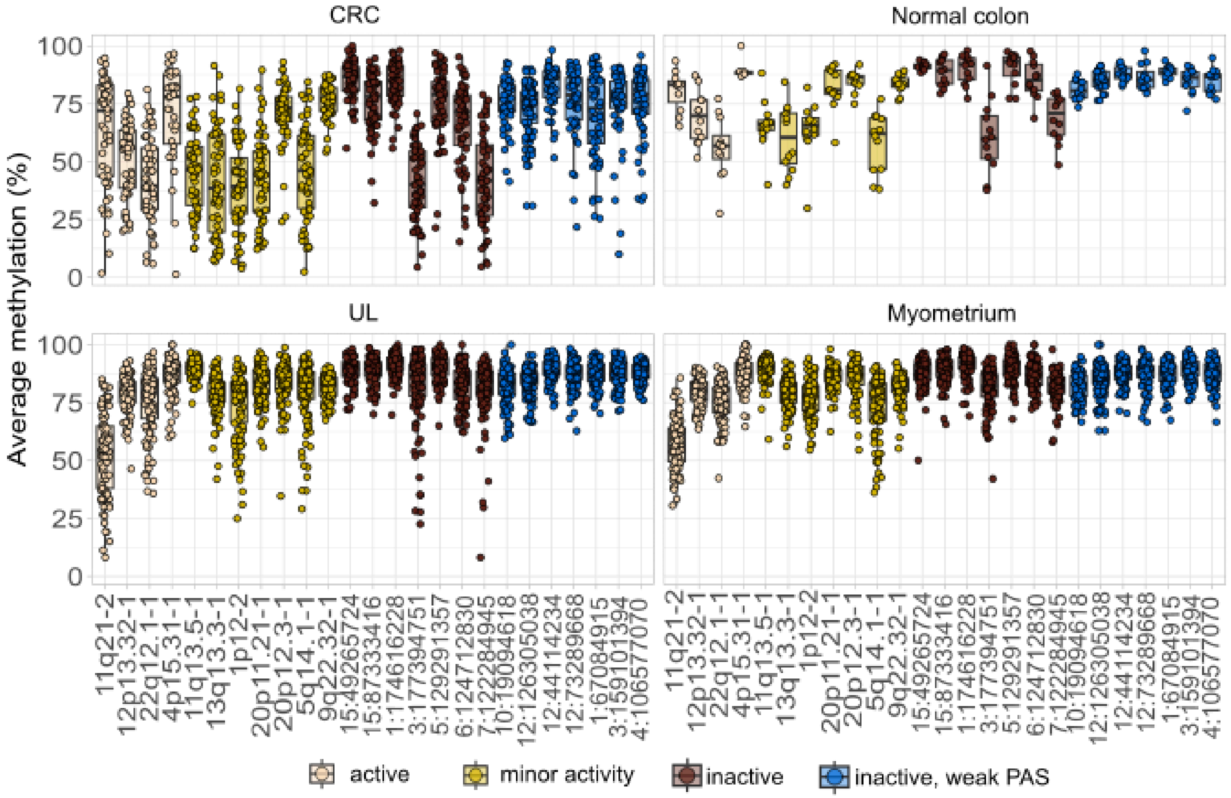

Average methylation levels from L1 5´ areas (200bp) for individual L1 elements of different activity types. Average methylation levels are shown for all active L1 elements having methylation data for 75% of the samples (n=4 L1 elements; beige) and for all L1 elements with weak polyadenylation signals (n=7 L1 elements; blue). The data is shown for 7 random L1 elements for L1s with minor activity (n = 7; yellow) or no activity (n = 7; brown). L1s with activity are named for their cytobands and coordinates are found in Supplementary Data S1. L1s with no activity are marked by the starting coordinates in reference genome hg38.

Supplementary Data

Supplementary Data S1: Active sources

Source elements that show somatic or germline transductional activity in either Nanopore or tag based detection. Contains information of their genomic contents, presence in samples and reference, and activity in Nanopore, tag based, and xTea detection. Additionally contains information of activity in two major transductions studies [2,3].

Supplementary Data S2: Somatic insertion in all detection

All somatic transductions detected with xTea, Nanopore and tag based detection. Every row contains information of a somatic transduction and whether it is detected by any of the detection methods. It is also detailed in the table if the transduction is detected by either Nanopore or xTea as a solo-L1 (this does not include tag based detection, as it detects only transductions). For every insertion, there is information on the insertion locus, source L1, and sample where detected.

Supplementary Data S3: Somatic activity in samples

Nanopore sequenced CRC samples and number of somatic insertions and transductions detected in them. Contains also the number of active source L1s detected in the tumors and the sequencing coverage as the number of Gbp mapped.

Supplementary Data S4: Somatic insertion features

Information of individual somatic transduction events, containing information of source L1 element, transduction breakpoints, insertion class, and hallmarks of retrotransposition.

Supplementary Data S5: Validation

Details of PCR validation of 20 somatic Nanopore calls and 20 somatic tag based calls. The table will display information on two types of PCR experiments and the sequencing of the products with either Sanger sequencing or Nanopore sequencing. Finally, the table concludes if any of the methods utilized was able to validate the insertion (insertion sequence and target sequence present), transduction (unique transduction sequence present) and the somatic nature of it (no band in the corresponding normal detected in the gel image of the PCR even though band in the insertion).

Supplementary Data S6: Insertion lengths

Lengths (combined lengths of L1 sequence, transduction sequence, and polyA-tail) of somatic and germline L1 insertions in Nanopore data. Contains information on all classes of insertions: solo-L1s, partnered transductions and orphan transductions.

Supplementary Data S7: Insertion classes

Insertion features in different insertion classes: (solo-L1s, partnered transductions and orphan transductions). Insertions are also divided into somatic and germline insertions. Contains the number of insertions, median length, and 5´inversion rate.

Supplementary Data S8: Polyadenylation signal downstream of source L1s

Presence of polyadenylation signals (PAS) downstream of the active source L1 elements. Contains the PAS distance (in bp) from the source L1, the PAs sequence and its strength.

Supplementary references

1. Nam, C. H. *et al.* Widespread somatic L1 retrotransposition in normal colorectal epithelium. *Nature* 1–8 (2023).
2. Rodriguez-Martin, B. *et al.* Pan-cancer analysis of whole genomes identifies driver rearrangements promoted by LINE-1 retrotransposition. *Nat. Genet.* **52**, 306–319 (2020).
3. Tubio, J. M. C. *et al.* Mobile DNA in cancer. Extensive transduction of nonrepetitive DNA mediated by L1 retrotransposition in cancer genomes. *Science* **345**, 1251343 (2014).
