# Supplementary material for: Nanopore sequencing reveals hidden landscape of short L1 transductions in colorectal cancer
Source: Commun Biol. 2026 Feb 12;9:418. doi: 10.1038/s42003-026-09674-z (PMC13009371; doi:10.1038/s42003-026-09674-z)
Supplement: Supplementary file 2 — Description of Additional Supplementary Files [file 42003_2026_9674_MOESM2_ESM.docx]

**Description of Additional Supplementary File**

File name: Supplementary Data

Description: The source data behind the graphs in the paper
